# Profiling endogenous airway proteases and antiproteases and modeling proteolytic activation of Influenza HA using *in vitro* and *ex vivo* human airway surface liquid samples

**DOI:** 10.1371/journal.pone.0306197

**Published:** 2024-12-31

**Authors:** Stephanie A. Brocke, Boris Reidel, Camille Ehre, Meghan E. Rebuli, Carole Robinette, Kevin D. Schichlein, Christian A. Brooks, Ilona Jaspers

**Affiliations:** 1 Curriculum in Toxicology and Environmental Medicine, University of North Carolina, Chapel Hill, NC, United States of America; 2 Marsico Lung Institute, University of North Carolina, Chapel Hill, NC, United States of America; 3 Center for Environmental Medicine, Asthma, and Lung Biology, University of North Carolina, Chapel Hill, NC, United States of America; 4 Department of Pediatrics, School of Medicine, University of North Carolina, Chapel Hill, NC, United States of America; 5 Department of Microbiology and Immunology, School of Medicine, University of North Carolina, Chapel Hill, NC, United States of America; University of South Dakota, UNITED STATES OF AMERICA

## Abstract

Imbalance of airway proteases and antiproteases has been implicated in diseases such as COPD and environmental exposures including cigarette smoke and ozone. To initiate infection, endogenous proteases are commandeered by respiratory viruses upon encountering the airway epithelium. The airway proteolytic environment likely contains redundant antiproteases and proteases with diverse catalytic mechanisms, however a proteomic profile of these enzymes and inhibitors in airway samples has not been reported. The objective of this study was to first profile extracellular proteases and antiproteases using human airway epithelial cell cultures and *ex vivo* nasal epithelial lining fluid (NELF) samples. Secondly, we present an optimized method for probing the proteolytic environment of airway surface liquid samples (*in vitro* and *ex vivo*) using fluorogenic peptides modeling the cleavage sites of respiratory viruses. We detected 48 proteases in the apical wash of cultured human nasal epithelial cells (HNECs) (n = 6) and 57 in NELF (n = 13) samples from healthy human subjects using mass-spectrometry based proteomics. Additionally, we detected 29 and 48 antiproteases in the HNEC apical washes and NELF, respectively. We observed large interindividual variability in rate of cleavage of an Influenza H1 peptide in the *ex vivo* clinical samples. Since protease and antiprotease levels have been found to be altered in the airways of smokers, we compared proteolytic cleavage in *ex vivo* nasal lavage samples from male/female smokers and non-smokers. There was a statistically significant increase in proteolysis of Influenza H1 in NLF from male smokers compared to female smokers. Furthermore, we measured cleavage of the S1/S2 site of SARS-CoV, SARS-CoV-2, and SARS-CoV-2 Delta peptides in various airway samples, suggesting the method could be used for other viruses of public health relevance. This assay presents a direct and efficient method of evaluating the proteolytic environment of human airway samples in assessment of therapeutic treatment, exposure, or underlying disease.

## Introduction

The human genome encodes 703 enzymes with known proteolytic activity and 1,652 endogenous antiproteases [1] which combined makes up over 10% of all protein coding genes in humans. Proteases and their endogenous antiproteases are fundamental to cellular homeostasis [2] and are expressed intracellularly, as membrane-bound proteins, and secreted extracellularly [3]. The mucosal surface of the airway represents a complex proteolytic landscape which must interact with environmental pathogens, allergens, and pollutants. There is evidence that the epithelial surface expresses a multitude of proteases with diverse catalytic mechanisms [4–8], but to our knowledge few studies have profiled proteases in the airway [9, 10], and none have specifically profiled antiproteases.

Proteases and antiproteases are important for cell- and tissue-level homeostasis and physiologic function. In the airways, a balance of proteases and antiproteases is necessary for bronchoconstriction [11–13], regulation of mucins and airway surface liquid [14–16], cell differentiation [17], development [18], repair, regeneration [19, 20], and immunity [21, 22]. Therefore, many pulmonary pathologies result from aberrant protease elevation or antiprotease depletion which perturb this complex proteolytic activity balance. The role of proteases in manifestation of respiratory diseases has been reviewed previously [23, 24].

Increased susceptibility to viral infection is one outcome of aberrant proteolytic activity in the respiratory tract [25]. To initiate infection, viral fusion proteins such as hemagglutinin (HA) in Influenza viruses and the Spike (S) protein in coronaviruses must be cleaved. This cleavage triggers activation of the viral fusogenic machinery, enabling fusion of the host and viral membranes and deposition of genetic material into the cytosol of the host cell. Respiratory viruses have evolved to commandeer extracellular and membrane-bound proteases expressed along the respiratory tract to cleave their fusion proteins and activate infection [26–28]. The HA subtypes in strains of mammalian Influenza A which continue to circulate in the human population, including H1, H2 and H3, are cleaved by a number of airway proteases: human airway trypsin-like protease (HAT) [29], matriptase [30, 31], kallikrein-related proteases [32], transmembrane serine protease 2 (TMPRSS2) [29, 33, 34], and plasmin [35]. The S protein of SARS coronaviruses is also cleaved by some of the same proteins [36–39]. Because of the role these proteases play in pathogenesis of viral infection, the therapeutic use of antiproteases as a prophylactic measure against infection has been extensively investigated [36, 40–44]. Furthermore, environmental exposures such as cigarette smoke and ozone have been found to alter secretion of proteases and antiproteases in the airways [45–47]. However, the effect of these alterations on susceptibility to viral infections remains understudied.

Proteases in the airway overlap in their substrate specificities, as exemplified above, and this is especially true within enzyme clans, which are designated based on mechanism of catalysis. Furthermore, endogenous inhibitors of proteases do not inhibit proteases of the same clan with equal efficacy [48]. Predicting general proteolytic activity of airway surfaces by protein detection or quantification methods is therefore difficult due to the abundance, diversity, and redundancy of proteases and antiproteases expressed there. Evaluation of Influenza HA cleavage by airway proteases has been used to approximate or determine susceptibility to infection using two general methods: (i) use of fluorogenic substrates modeling the Influenza HA cleavage site to investigate cleavage by individual proteases [35, 49], and (ii) use of Western blotting to detect HA cleavage fragments by individual enzymes or cell culture supernatants [31, 46, 50, 51]. Previously, using organotypic human nasal epithelial cell (HNEC) cultures, we validated that apically-secreted proteases cleave the HA protein of intact H1N1 influenza virus during infection via Western blot [46]. Furthermore, our experiments showed that immunoprecipitation of specific proteases (i.e. TMPRSS2 and HAT) from apical wash samples prior to incubation with H1N1 influenza virus resulted in decreased cleavage of the HA protein, confirming that extracellular proteases secreted by differentiated airway cultures cleave viral fusion proteins.

In the present study, we sought to first profile the diverse and abundant proteases and antiproteases secreted from *in vitro* primary airway epithelial cells grown at air-liquid interface (ALI) as well as *ex vivo* nasal epithelial lining fluid (NELF) from healthy human donors. Next, we present a novel methodology for probing the proteolytic activity of these samples toward Influenza H1 using a non-infectious internally quenched fluorescent (IQF) peptide modelled after the viral cleavage site (Table 1). We also demonstrate utility of this assay towards additional viral substrates, SARS-CoV-1 S and SARS-CoV-2 S. Using organotypic cultures of nasal and bronchial epithelial cells, we compared proteolytic activation of the viral peptides in multiple airway regions. This method offers a more high-throughput and straightforward approach to assessing susceptibility to Influenza infection relative to immunoassay- or gel-based detection methods which also require larger sample volumes. Comparison of proteolytic activation of viral substrates pre- and post-environmental exposure, in potentially susceptible groups, or as a screen for efficacy of preventative therapeutics represent applications of this method. To this end, we evaluated rates of Influenza H1 cleavage in previously collected nasal lavage fluid (NLF) from smokers and non-smokers and demonstrate its utility even with extremely non-invasive NELF samples.

**Table 1:** Amino acid sequences of peptides used for experimentation and residue numbers from the full-length proteins.

Peptide	Amino acid residues	Sequence (N’-C’)	N’-terminus modification	C’-terminus modification	GenBank Accession
**Influenza HA A/California/04/2009 H1N1**	338–346	IPSIQS**RG**L	MCA	Lysine-DNP	ACP41105
**SARS-CoV-1 S wild type**	661–671	HTVSLL**RS**TSQ	MCA	Lysine-DNP	ABD73002
**SARS-CoV-2 S wild type**	678–688	TNSPRRA**R****S**VA	MCA	Lysine-DNP	QIG55955
**SARS-CoV-2 S Delta variant**	678–688	TNSRRRA**R****S**VA	MCA	Lysine-DNP	UAL04647

For each peptide, the site of cleavage occurs between the two residues highlighted in red. The SARS coronavirus peptides model the S1/S2 cleavage site. The multi-basic Furin cleavage site insertion acquired by SARS-CoV-2 is underlined. MCA = 7-methoxycoumarin-4-yl acetyl, DNP = N-2,4-dinitrophenyl.

## Materials and methods

### Culture of HNECs and HBECs

Primary human nasal epithelial cells (HNECs) were obtained from healthy adult volunteers aged 18–59 years. Demographic information of HNEC donors is provided (Table 2). Subjects were recruited between November 1, 2019 and March 11, 2021 by a protocol approved by the Institutional Review Board at the University of North Carolina (Protocol # IRB 11–1363). Written informed consent was obtained from all subjects. Complete sample collection and culturing methods have been published previously [52], but briefly, inferior turbinate nasal scrapes were collected from each donor and expanded in flasks using PneumaCult-Ex Plus Medium (STEMCELL, Vancouver, BC, Canada) supplemented with 1% penicillin-streptomycin at 5% CO_2_ and 37°C. After two passages, cells were frozen down using Bambanker medium (Lymphotec, Tokyo, Japan) and stored in liquid nitrogen. For experimentation, cells were thawed and seeded in a flask for one additional expansion. Upon confluency, cells were seeded onto permeable polyethylene terephthalate 12-well inserts with a 0.4 μm pore size (CELLTREAT, Pepperell, MA, USA) which were coated with human placental type IV collagen (Sigma-Aldrich, St. Louis, MO; C7521). Ex Plus medium was added to both the apical and basolateral compartments and changed daily until the cells reached confluency on the inserts. At confluency, Ex Plus medium was exchanged for PneumaCult ALI medium (STEMCELL, Vancouver, BC, Canada) on the basolateral side and medium was removed on the apical side. For one week, basolateral medium was replaced daily. From then on, three times per week the medium was changed, and the apical surface was washed with Hanks Balanced Saline Solution +CaCl_2_, +MgCl_2_, (HBSS++). Cells were differentiated at ALI conditions for >30 days, until ciliation and mucus were present on the cultures.

**Table 2:** Demographic information of HNEC donors used for proteomic analysis (n = 6) as well as HNEC (n = 13) and HBEC (n = 3) donors used for proteolytic cleavage assays.

	HNECs for proteomics (n = 6)	HNECs (n = 13)	HBECs (n = 3)
Age, mean±standard deviation	48.5±13.9	26.9±6.1	36.7±4
Sex, n, M/F	4/2	8/5	2/1
Race, n, Black/White/Asian	0/6/0	1/10/2	1/2/0
Ethnicity, n, Hispanic/Non-Hispanic	1/5	2/11	0/3

Primary human bronchial epithelial cells (HBECs) were sourced from non-diseased, non-smoker adult donors through the Marsico Lung Institute Tissue Procurement and Cell Culture Core at the University of North Carolina. Demographic data are provided in Table 2. Cells were cultured and differentiated as described previously [53, 54]. The cells were initially cultured in flasks coated with bovine type-1 collagen (Advanced BioMatrix, Carlsbad, CA, USA) in PneumaCult-Ex Plus medium (STEMCELL, Vancouver, BC, Canada) supplemented with 1% penicillin-streptomycin. Upon reaching 70–90% confluence, the cells were passaged with Accutase (Innovative Cell Technologies, San Diego, CA, USA). Passage 3 cells were plated on 12 mm, 0.4-μm permeable cell culture inserts (CELLTREAT, Pepperell, MA, USA) coated with human placental type IV collagen (Sigma-Aldrich, St. Louis, MO, USA). The cells were cultured using Ex Plus in both the basolateral and apical compartments for 1 week. Upon achieving confluence, the cells were subjected to differentiation at an air-liquid interface (ALI) for at least 30 days with Pneumacult ALI (STEMCELL, Vancouver, BC, Canada) growth medium in the basolateral compartment. Basolateral media was initially replaced daily for 1 week, then subsequently replaced three times a week, and the apical surface was washed once a week with HBSS++ to clear apical mucus and cellular debris.

### HNEC and HBEC sample collection

Once cells were differentiated, apical washes for proteolytic cleavage assays were collected by adding 200 μl of 37°C HBSS++ to the apical surface of each culture and incubating at room temperature for 15 minutes. Apical wash liquid was then carefully removed with a pipette and pooled by donor in microcentrifuge tubes. Samples were stored at -80°C.

### Nasal lavage fluid (NLF) sample collection

NLF was collected and processed from healthy adults aged 18–50 years who were either never-smokers or cigarette smokers defined based on self-reporting and usage of smoking diaries, as described previously [55, 56]. Samples were collected between August 1, 2014 and March 1, 2016. Exclusion criteria included symptoms of allergic rhinitis, chronic obstructive pulmonary disease, asthma, and use of immunosuppressive drugs such as corticosteroids. Demographic data from these participants are provided in Table 3. The protocol used for NLF sample collection was approved by the University of North Carolina’s Institutional Review Board (Protocol # IRB 11–1363) and written informed consent was obtained from all subjects.

**Table 3:** Demographic information of NLF donors.

	All (n = 48)	Cigarette smokers (n = 24)	Non-smokers (n = 24)
Age, mean±standard deviation	30.1±6.7	30.8±6.4	29.4±6.9
Sex, n, M/F	24/24	12/12	12/12
Race, n, Black/White/Asian	16/31/1	12/11/1	4/20/0

### Nasal epithelial lining fluid (NELF) sample collection

NELF was collected as previously described [57] on October 26, 2023 from n = 19 donors by a protocol approved by the Institutional Review Board at the University of North Carolina (Protocol # IRB 11–1363) and written informed consent was obtained from each subject. Demographic information for sample donors is provided in Table 4. Briefly, nostrils were sprayed with 0.9% saline solution, then Leukosorb paper (Pall Scientific, Port Washington, NY, USA) cut in strips to fit the nostrils were inserted into both nares. A padded clip was used to clamp the nostrils closed and keep the Leukosorb strips in place for 2 minutes. The Leukosorb strips were removed and stored in 1.5 ml microcentrifuge tubes at -20°C until elution. Eluate from the Leukosorb strips was collected as previously described [57]. A 100 μl volume of 1% BSA + 0.05% Triton X-100 in PBS was pipetted onto each strip. The strips were then centrifuged twice at 13,000 rpm for 2 minutes to collect the eluate at the bottom of the tube.

**Table 4:** Demographic information of NELF donors.

	All (n = 19)
Age, mean±standard deviation	29.9±6.4
Sex, n, M/F	10/9
Race, n, Black/White/Asian	3/13/3
Hispanic/Latino, n, Y/N	3/16

### Mass spectrometry-based proteomic analysis

HNEC culture apical secretions and nasal strip samples were prepared for label-free proteomics using filter-aided sample preparation (FASP) [58]. HNEC apical washes from n = 6 donors (4M, 2F) and NELF from n = 13 donors (5M, 8F) were used for proteomics. For HNEC secretions 100 μl apical washes were denatured using 8M urea, and nasal strips were extracted in 0.5 ml 4M GuHCl, followed by the reduction of cysteine residues by 10 mM dithiothreitol (Sigma-Aldrich, St. Louis, MO, USA) and alkylation in 50 mM iodoacetamide (Sigma-Aldrich). Samples were digested with pig trypsin (25 ng/μl) overnight at 37°C. The resulting peptide mixtures were vacuum freeze-dried and dissolved in 30 μl of 1% acetonitrile and 0.1% trifluoroacetic acid, and 5 μl were injected into each sample for chromatography tandem mass spectrometry (LC-MS/MS). LC-MS/MS analysis was performed utilizing a Q-Exactive (Thermo Scientific, Waltham, MA, USA) mass spectrometer coupled to an Ultimate 3000 nano HPLC system (Thermo Scientific), and data acquisitions were performed as described previously [59]. The raw data were processed and searched against the UniProt protein database (Homo sapiens, November 2023) using Proteome Discoverer 1.4 (Thermo Scientific, Waltham, MA, USA) software. The following parameters were used in the Sequest search engine: 10 ppm mass accuracy for parent ions and 0.02 Da accuracy for fragment ions; 2 missed cleavages were allowed. The carbamidomethyl modification for cysteines was set to fixed, and methionine oxidation was set to variable. Scaffold 5.3.0 (Proteome Software Inc., Portland, OR, USA) was used to validate the MS/MS-based peptide and protein identifications. Peptide identifications were accepted if they had greater than 95.0% probability by the scaffold local FDR algorithm. Protein identifications were accepted if they had greater than 99.0% probability and contained at least 2 identified peptides. Protein probabilities were assigned by the Protein Prophet algorithm [60]. Proteins that contained similar peptides and could not be differentiated based on MS/MS analysis alone were grouped to satisfy the principles of parsimony. The resulting protein identification lists were annotated using NCBI annotations and filtered using the Gene Ontology Terms “endopeptidase activity” (GO:0004175) plus “exopeptidase activity” (GO:0008238) for the protease lists, and “peptidase inhibitor activity” (GO:0030414) for the protease inhibitor lists.

### Viral IQF peptide design

The design of the IQF peptides was based on the work by Jaimes and Straus et al. [35, 37]. The amino acid sequences of all IQF peptides used in our assays are provided in Table 1. An additional peptide modeled after the SARS-CoV-2 Delta variant was also constructed because of its highly conserved P681R mutation in the cleavage site. This mutation is believed to aid in viral fusion with the host and render the strain more pathogenic than the wild-type strain [61]. The peptides were modified to include the fluorophore 7-methoxycoumarin-4-yl acetyl (MCA) on the N-terminus and an additional Lysine residue with N-2,4-dinitrophenyl (DNP) as a quencher on the C-terminus. Custom peptides were ordered from Biomatik (Kitchener, Ontario, Canada) and arrived as 1 mg aliquots of lyophilized powder and stored at -20°C. Peptides were ordered with the following specifications; purity >95%, TFA removed, and switched to HCl salt. Before use, the peptides were brought to room temperature then resuspended in pure dimethyl sulfoxide (DMSO) at 1 mg/ml. This concentrated peptide stock was then aliquoted and stored at -80°C.

### Proteolytic cleavage assay protocol

Assays were performed in half-area black 96-well plates (Corning Inc, Corning, NY, USA) with a total reaction volume of 50 μl. Airway culture samples and *ex vivo* airway samples were thawed on ice, vortexed briefly and centrifuged at 8,000 x g for 2 minutes to collect mucus and debris. For each assay, 25 μl of sample was used unless otherwise indicated. The frozen peptide stock aliquots were thawed and diluted to 250 μM in PBS with 25% DMSO. Then 10 μl of diluted peptide was added to each well for a final assay peptide concentration of 50 μM, 5% DMSO. PBS was added for its buffering capacity to bring the total assay volume to 50 μl. A multichannel pipet was used to add the dilute peptide to the assay plate immediately prior to assay initiation to increase substrate loading efficiency. A mixture of protease inhibitors was used when indicated, which consisted of 33 μM camostat mesylate (a serine protease inhibitor [62]) (Bio-Techne, Minneapolis, MN, USA), 33 μM decanoyl-RVKR-Chloromethylketone (a proprotein convertase inhibitor [63]) (Bio-Techne, Minneapolis, MN, USA), and 33 μM E64 (a cysteine protease inhibitor [64]) (Sigma-Aldrich, St. Louis, MO, USA). Additionally, recombinant human Furin (New England Biolabs Inc., Ipswich, MA, USA) was diluted in PBS and added to assays as indicated at a concentration of 10 U/ml. Fluorescence was measured with an excitation wavelength of 330 nm and emission wavelength of 390 nm using a CLARIOstar Plus plate reader (BMG Labtech, Ortenburg, Germany). Fluorescence intensity from each well was averaged across 16 locations in the well and was measured approximately once every 1–2 minutes (dependent upon total cycle length) for 60 cycles. The gain setting remained constant for all assays. For all data shown, maximum of slope measurements were based on a width of 6 minutes.

### Proteolytic cleavage assay on ALI culture surface

Proteolytic activity was measured directly on the apical surface of two replicate cultures from n = 3 HBEC donors. Immediately prior to the assay, 100 μl of assay solution with 50 μM peptide and 5% DMSO in PBS were pipetted onto the apical surface of the cultures growing on CELLTREAT culture inserts. The plate was then read in a CLARIOstar plate reader as described above, but an Atmospheric Control Unit was additionally used to maintain the assay chamber at 37°C and 5% CO_2_ for the duration of the assay to maintain culture quality. Assays were conducted in clear, 12-well cell culture plates (Corning Inc, Corning, NY, USA) with inserts in place and 1 ml of Pneumacult ALI medium in the basolateral compartment.

### Statistical analysis

Statistical tests and data visualization were performed using GraphPad Prism software (v 10.2.0). ANOVA and *t* tests were used to test for differences between groups and paired/repeated measures versions of the tests were implemented where appropriate. Bonferroni’s post hoc test was used in combination with ANOVA. P values ≤ 0.05 were considered statistically significant.

## Results

### Proteomic profiling of proteases and antiproteases in human airway samples

The landscape of proteases and antiproteases present in human nasal epithelial cell (HNEC) apical washes and nasal epithelial lining fluid (NELF) samples from healthy donors was evaluated with mass spectrometry-based proteomics. The proteases detected uniquely in each sample type as well as in both sample types are shown in Table 5, likewise with antiproteases in Table 6. A greater number of proteases and antiproteases were detectable in NELF samples (57 and 48 respectively) compared to HNEC apical wash (48 and 29). There were 26 proteases and 19 antiproteases present in both sample types. Enzymes with a variety of catalytic mechanisms were detected, including cysteine proteases, serine proteases, and metalloproteases. Accordingly, protease inhibitors against multiple catalytic mechanisms were also detected; cystatins (cysteine protease inhibitors), SERPINs (serine protease inhibitors), TIMPs (tissue inhibitors of metallopeptidases), and others.

**Table 5:** List of proteases detected uniquely in HNEC apical wash samples (n = 6, 4M, 2F) or NELF samples (n = 13, 5M, 8F) and in both sample types by mass spectrometry based proteomic analysis.

**Proteases found in both sample types**		
**Proteases**	**Full name**	**Catalysis type**
ANPEP	alanyl aminopeptidase	metallo
APEH	acylpeptide hydrolase	serine
CAPN1	calpain-1	cysteine
CAPN2	calpain-2	cysteine
CAPNS1	calpain small subunit 1	cysteine
CFI	complement factor I	serine
CLCA4	chloride channel accessory 4	metallo
CNDP2	carnosine dipeptidase II	metallo
CTSB	cathepsin B	cysteine
CTSC	cathepsin C / dipeptidyl peptidase I	cysteine
CTSD	cathepsin D	aspartic
CTSH	cathepsin H	cysteine
CTSS	cathepsin S	cysteine
CTSV	cathepsin V	cysteine
DPP3	dipeptidyl peptidase 3	metallo
KLK10	kallikrein related peptidase 10	serine
KLK11	kallikrein related peptidase 11	serine
KLK13	kallikrein related peptidase 13	serine
LAP3	leucine aminopeptidase 3	metallo
LTA4H	leukotriene A4 hydrolase	metallo
MMP9	matrix metallopeptidase 9	metallo
NPEPPS	cytosol alanyl aminopeptidase	metallo
PIP	prolactin-induced protein	aspartic
PSMB8	proteasome subunit beta type-8	threonine
RNPEP	aminopeptidase B	metallo
XPNPEP1	X-prolyl aminopeptidase 1	metallo
**Proteases unique to HNEC apical washes (n = 6)**		
**Proteases**	**Full name**	**Catalysis type**
ACE	angiotensin-I-converting enzyme	metallo
ADAM10	a disintegrin and metalloproteinase domain-containing protein 10	metallo
ADAM28	a disintegrin and metalloproteinase domain-containing protein 28	metallo
ADAM9	a disintegrin and metalloproteinase domain-containing protein 9	metallo
AGA	aspartylglucosaminidase	threonine
CAPN5	calpain-5	cysteine
CFD	complement factor D	serine
CTSA	cathepsin A	serine
CTSZ	cathepsin X	cysteine
DPP7	dipeptidyl peptidase 7	serine
GGH	gamma glutamyl hydrolase	cysteine
KLK6	kallkrein related peptidase 6	serine
MMP10	matrix metallopeptidase 10	metallo
NCSTN	nicastrin	metallo
NPEPL1	leucyl aminopeptidase like 1	metallo
PRSS22	serine protease 22 / prosemin	serine
PRSS8	prostatin	serine
PSMB6	proteasome subunit beta type-6	threonine
PSMB9	proteasome subunit beta type-9	threonine
SCPEP1	serine carboxypeptidase 1	serine
ST14	transmembrane serine protease matriptase	serine
TMPRSS11E	transmembrane serine protease 11E	serine
**Proteases unique to NELF (n = 13)**		
**Proteases**	**Full name**	**Catalysis type**
ASPRV1	aspartic peptidase retroviral like 1 / skin aspartic protease	aspartic
ASRGL1	asparaginase / isoaspartyl peptidase 1	threonine
BLMH	bleomycin hydrolase	cysteine
C1R	complement C1r	serine
C1S	complement C1s	serine
C2	complement C2	serine
CASP14	capase 14	cysteine
CASP3	caspase 3	cysteine
CFB	complement factor B	serine
CPA4	carboxypeptidase A4	metallo
CPD	carboxypeptidase D	metallo
CPM	carboxypeptidase M	metallo
CTSG	cathepsin G	serine
ELANE	neutrophil elastase	serine
F12	coagulation factor XIIa	serine
F2	thrombin	serine
FOLH1B	folate hydrolase 1	metallo
HTRA1	serine protease HTRA1	serine
IDE	insulin degrading enzyme / insulysin	metallo
KLK1	kallikrein 1	serine
KLK14	kallikrein related peptidase 14	serine
KLK7	kallikrein related peptidase 7	serine
KLK8	kallikrein related peptidase 8	serine
KLKB1	plasma kallikrein	serine
MMP8	matrix metallopeptidase 8	metallo
PLG	plasmin	serine
PRTN3	proteinase 3 / myeloblastin	serine
PSMB5	proteasome subunit beta type-5	threonine
TMPRSS11D	human airway trpysin-like protease	serine
TPP1	tripeptidyl peptidase 1	serine
USP5	ubiquitin specific peptidase 5	cysteine

**Table 6:** List of antiproteases detected uniquely in HNEC apical wash samples (n = 6, 4M, 2F) or NELF samples (n = 13, 5M, 8F) and in both sample types by mass spectrometry based proteomic analysis.

**Antiproteases found in both sample types**		
**Antiproteases**	**Full name**	**Inhibitor of**
A2ML1	alpha-2-macroglobulin like 1	endopeptidases (all catalytic types)
C3	complement 3	endopeptidases (all catalytic types)
CST3	cystatin C	papain-like cysteine peptidases
CST6	cystatin E/M	papain-like cysteine peptidases
CSTA	cystatin A	papain-like cysteine peptidases
CSTB	cystatin B	papain-like cysteine peptidases
PEBP1	phosphatidylethanolamine binding protein 1	serine carboxypeptidase Y
PI3	peptidase inhibitor 3	serine endopiptidases
RARRES1	retinoic acid receptor responder protein 1	metallocarboxypeptidases
SERPINA1	alpha-1 antitrypsin	serine and cysteine endopeptidases
SERPINA3	serpin family A member 3	serine and cysteine endopeptidases
SERPINB1	leukocyte elastase inhibitor	serine and cysteine endopeptidases
SERPINB3	serpin family B member 3	serine and cysteine endopeptidases
SERPINB4	serpin family B member 4	serine and cysteine endopeptidases
SERPINB5	serpin family B member 5	serine and cysteine endopeptidases
SERPINF1	serpin family F member 1	serine and cysteine endopeptidases
SLPI	secretory leukocyte protease inhibitor	serine endopiptidases
THBS1	thrombospondin 1	serine peptidases
WFDC2	WAP four-disulfide core domain 2	serine endopiptidases
**Antiproteases unique to HNEC apical wash (n = 6)**		
**Antiproteases**	**Full name**	**Inhibitor of**
APP	amyloid precursor protein	serine peptidases
BST2	bone marrow stromal antigen 2	metalloendopeptidases
C4B	complement component 4B	endopeptidases (all catalytic types)
CD109	cluster of differentiation 109	endopeptidases (all catalytic types)
LXN	latexin	metallocarboxypeptidases
SERPINB2	plasminogen activator inhibitor-2	serine and cysteine endopeptidases
SERPINB6	serpin family B member 6	serine and cysteine endopeptidases
SPINK5	serine protease inhibitor Kazal-type 5	serine endopeptidases
SPINT1	serine peptidase inhibitor, Kunitz type 1	serine peptidases
TIMP2	tissue inhibitor of metallopeptidase 2	metalloendopeptidases
**Antiproteases unique to NELF (n = 13)**		
**Antiproteases**	**Full name**	**Inhibitor of**
A2M	alpha-2-macroglobulin	endopeptidases (all catalytic types)
AGT	angiotensinogen	serine and cysteine endopeptidases
AHSG	alpha 2-HS glycoprotein	papain-like cysteine peptidases
AMBP	alpha-1-microglobulin/Bikunin precusor	serine peptidases
C4A	complement component 4	endopeptidases (all catalytic types)
C5	complement c5	endopeptidases (all catalytic types)
CAST	calpastatin	calpains
CST1	cystatin SN	papain-like cysteine peptidases
CST2	cystatin SA	papain-like cysteine peptidases
CST4	cystatin S	papain-like cysteine peptidases
CST5	cystatin D	papain-like cysteine peptidases
HRG	histidine rich glycoprotein	papain-like cysteine peptidases
ITIH1	inter-alpha-trypsin inhibitor heavy chain 1	serine peptidases
ITIH2	inter-alpha-trypsin inhibitor heavy chain 2	serine peptidases
ITIH4	inter-alpha-trypsin inhibitor heavy chain 4	serine peptidases
KNG1	kininogen 1	papain-like cysteine peptidases
LCN1	lipocalin 1	calpains
LTF	lactotransferrin	serine peptidases
OPRPN	opiorphin prepropeptide	metalloendopeptidases
PZP	alpha-2-macroglobin like	endopeptidases (all catalytic types)
SERPINA4	serpin family A member 4	serine and cysteine endopeptidases
SERPINA7	serpin family A member 7	serine and cysteine endopeptidases
SERPINB10	serpin family B member 10	serine and cysteine endopeptidases
SERPINB12	serpin family B member 12	serine and cysteine endopeptidases
SERPINC1	antithrombin III	serine and cysteine endopeptidases
SERPINF2	alpha-2-antiplasmin	serine and cysteine endopeptidases
SERPING1	plasma protease C1 inhibitor	serine and cysteine endopeptidases
SMR3B	submaxillary gland androgen regulated protein 3B	predicted endopeptidase activity
TIMP1	tissue inhibitor of metalloproteinase 1	metalloendopeptidases

### Measuring protease activity in HNEC mucociliary surface secretions

**Fig 1:**
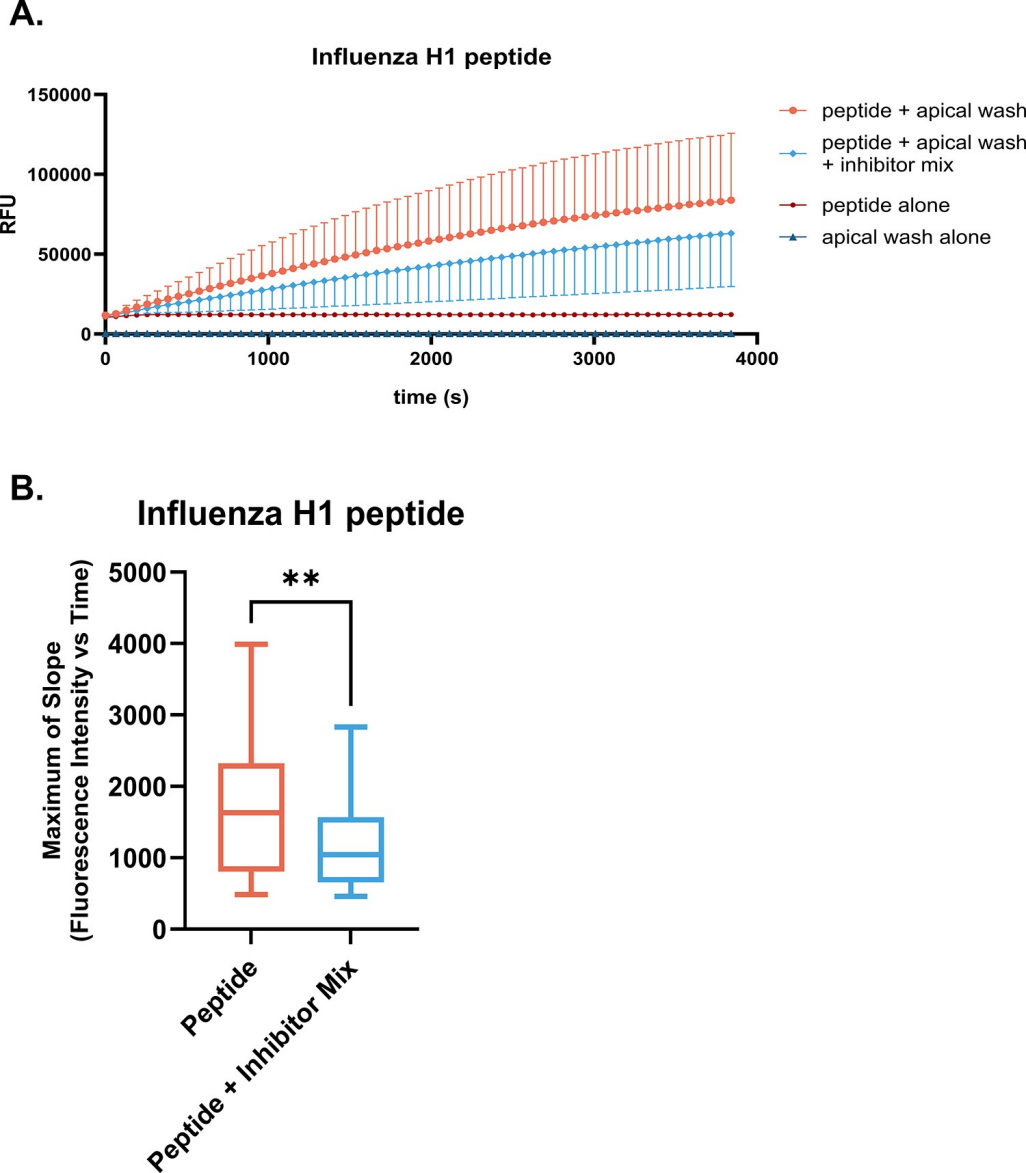
Proteases in HNEC apical wash cleave Influenza H1. HNECs from n = 9 (5M, 4F) donors were cultured at air liquid interface. Apical washes from differentiated cultures were assayed for proteolytic activity toward the influenza H1 peptide by mixing each sample with 50 μM of the peptide and measuring fluorescence intensity over time. A) The mean change in relative fluorescence units (RFU) versus time (s) for all donors is shown in orange. Each donor was assayed in triplicate. Addition of a protease inhibitor mix on rate of cleavage is indicated by the light blue line. Controls include the peptide at 50 μM without addition of HNEC apical wash (red) and HNEC apical wash alone, without the peptide (dark blue). Samples were collected 4 d since the prior apical wash. B) Maximum of slope of the cleavage reaction is plotted and a paired *t*-test was used to evaluate differences between groups; ** indicates p<0.01.

Apical secretions from differentiated HNECs from n = 9 (5M, 4F) donors were tested for proteolytic activity toward the Influenza H1 internally quenched fluorescent (IQF) peptide. Combining apical wash liquid with the IQF peptides resulted in an increase in fluorescence intensity over time relative to assaying the peptide alone at the same concentration (50 μM), shown in Fig 1A. Addition of a mixture of protease inhibitors against serine, cysteine, and Furin-like proteases resulted in a statistically significant reduction in the maximum slope of the cleavage reaction (change in fluorescence intensity versus time) of Influenza H1 (Fig 1B). Of note, there was a statistically significant difference in age of HNEC donors used for proteomics versus for proteolytic cleavage assays (p = 0.0419) using Brown-Forsythe and Welch’s ANOVA tests, with Dunnett’s post hoc test. For proteomic analysis, the mean age of HNEC donors was 48.5±13.9 years and the mean age of HNEC donors for proteolytic cleavage assays was 26.9±6.1 years.

After an initial wash at time = 0, apical washing with HBSS++ was repeated on separate cultures from multiple HNEC donors at 24, 48, 72, or 96h. S1 Fig demonstrates that proteolytic activity of HNEC apical secretions toward Influenza H1 increased with duration since the prior apical wash, albeit the change was not statistically significant. Furthermore, apical washes from n = 5 HNEC donors demonstrated stable proteolytic activity toward the Influenza H1 IQF peptide from zero to four freeze and thaw cycles (S2 Fig), suggesting feasibility of applying the proteolytic cleavage assay to stored samples.

### Maximum rate of cleavage increases with substrate concentration

**Fig 2:**
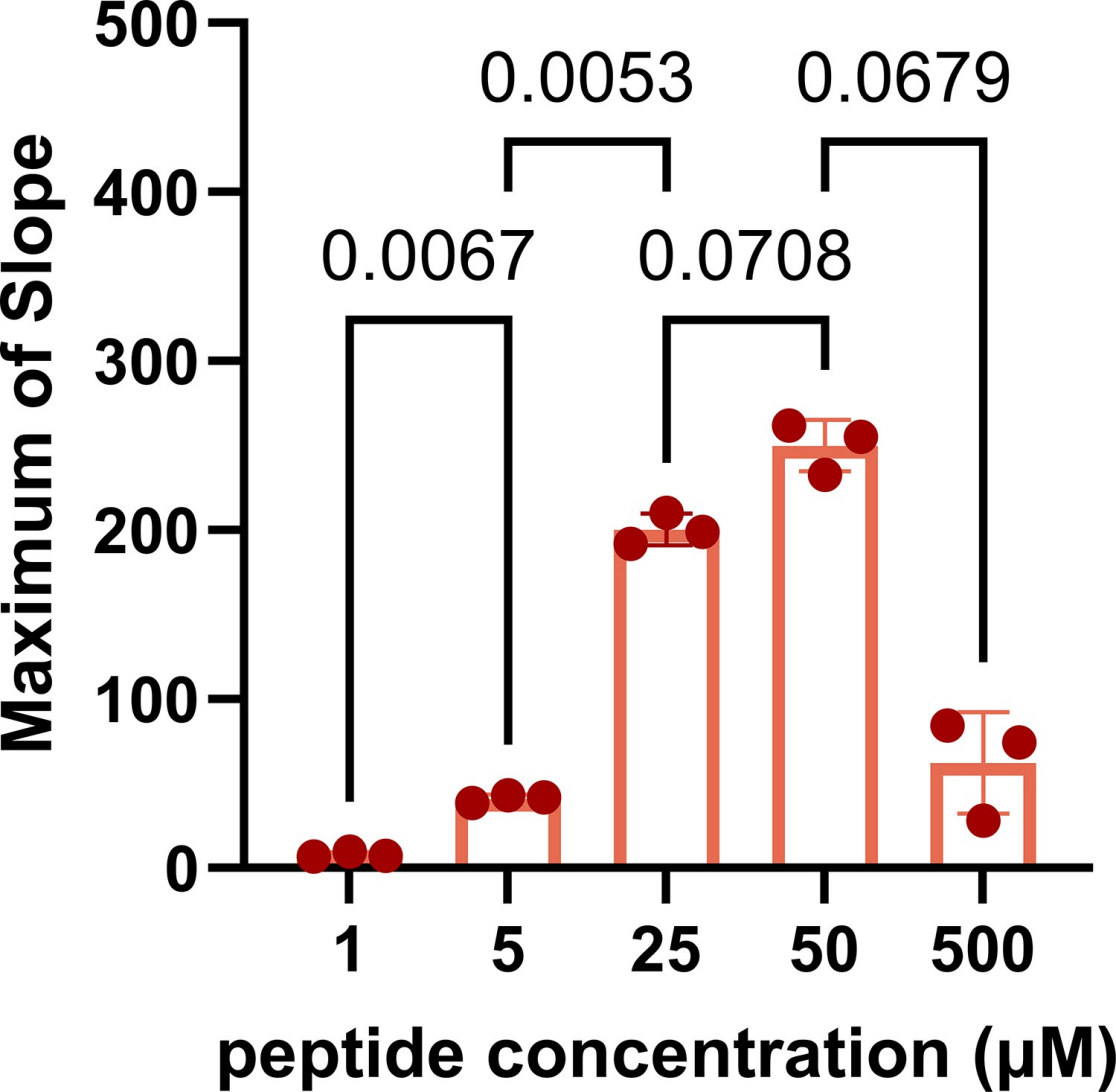
Rate of cleavage increases with substrate concentration up to 50 μM. Maximum rates of cleavage of Influenza H1 peptide at a range of concentrations from 1–500 μM tested with apical wash samples from n = 3 (2M, 1F) HNEC donors. A repeated measures one-way ANOVA with Bonferroni’s post hoc test was used to determine differences between groups, p≤0.05.

The impact of the Influenza H1 substrate concentration on the maximum slope of the cleavage reaction was evaluated. Maximum rates of cleavage with 1, 5, 25, 50, and 500 μM peptide concentrations were tested using apical washes from n = 3 HNEC donors. Maximum slope of the cleavage reaction increased with concentration up to 50 μM, and dropped off substantially at 500 μM (Fig 2), suggesting optimal saturation of the assay around 50 μM.

### Proteolytic activity toward Influenza H1 is detectable in multiple airway cell types

**Fig 3:**
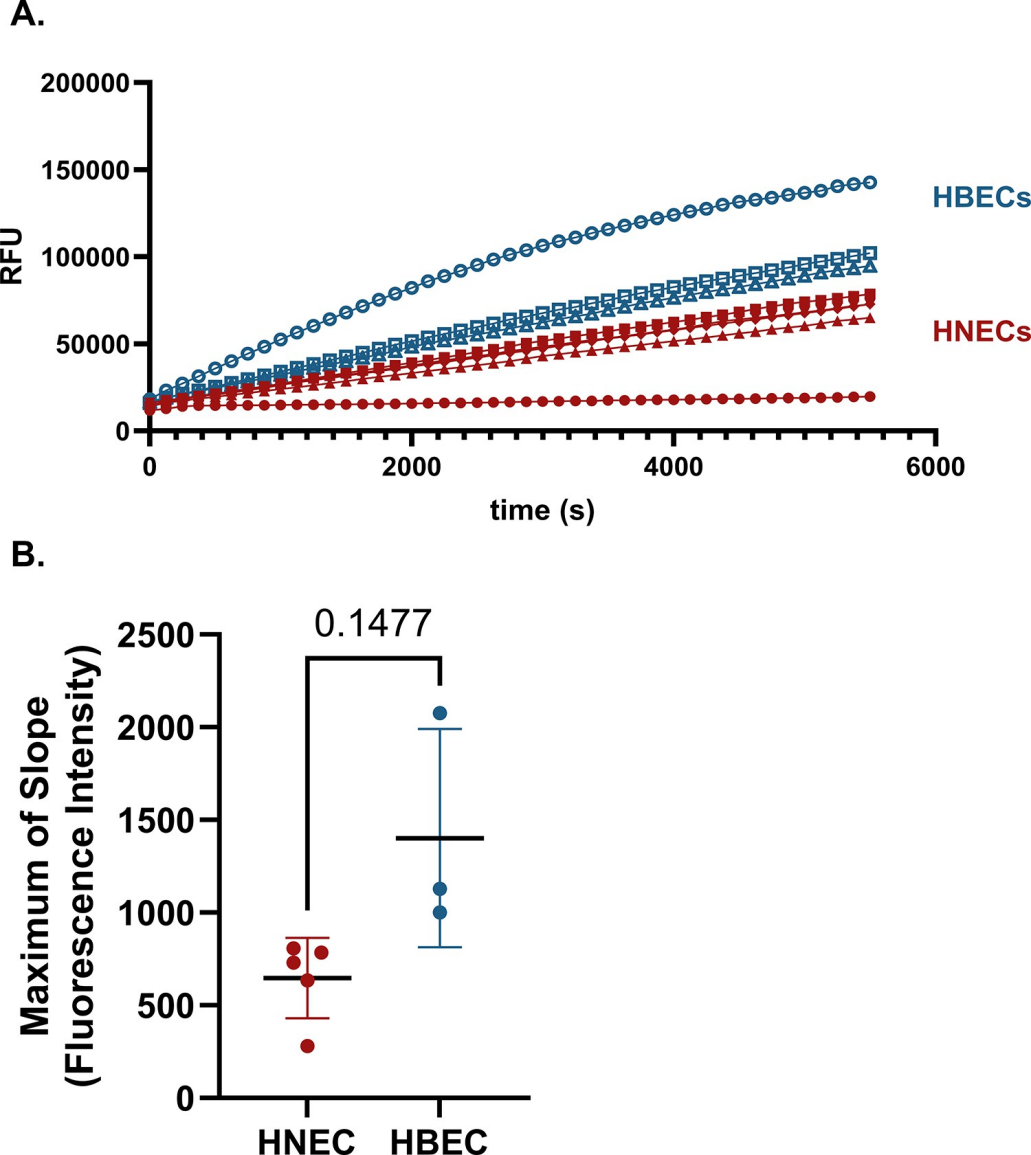
Comparison of Influenza H1 cleavage by proteases in nasal vs bronchial samples. A) HNEC and HBEC cultures grown at ALI were apically washed. Samples were then mixed with an influenza H1 IQF peptide and fluorescence intensity in each sample over time was measured in a microplate reader. Rates of cleavage of Influenza H1 by proteases in apical wash samples from n = 5 (3M, 2F) HNEC donors and n = 3 (2M, 1F) HBEC donors are shown, each line representing a single donor averaging three technical replicates with standard deviation. HBECs are shown in blue and HNECs are shown in red. All samples were collected 7 d from the prior apical wash. B) Maximum of slope of the cleavage reaction is plotted. A Welch’s unequal variances *t*-test was used to test for difference between groups.

Like HNECs, human bronchial epithelial cells (HBECs) are also commonly grown at air-liquid interface as an organotypic airway model system, and Influenza virus has been shown to replicate successfully in both the nasal and bronchial epithelium [65]. Proteolytic activity toward Influenza H1 was compared in apical washes from n = 5 HNEC (3M, 2F, mean age 25.7±3.3) and n = 3 (2M, 1F, mean age 36.7±4.0) HBEC donors, which were normalized to a total protein concentration of 58 ng/μl, shown in Fig 3. Lines represent individual donors, color coded by airway region. No statistically significant difference in cleavage of Influenza H1 was observed between the two airway cell types.

### The apical surface of ALI cultures is proteolytically active

**Fig 4:**
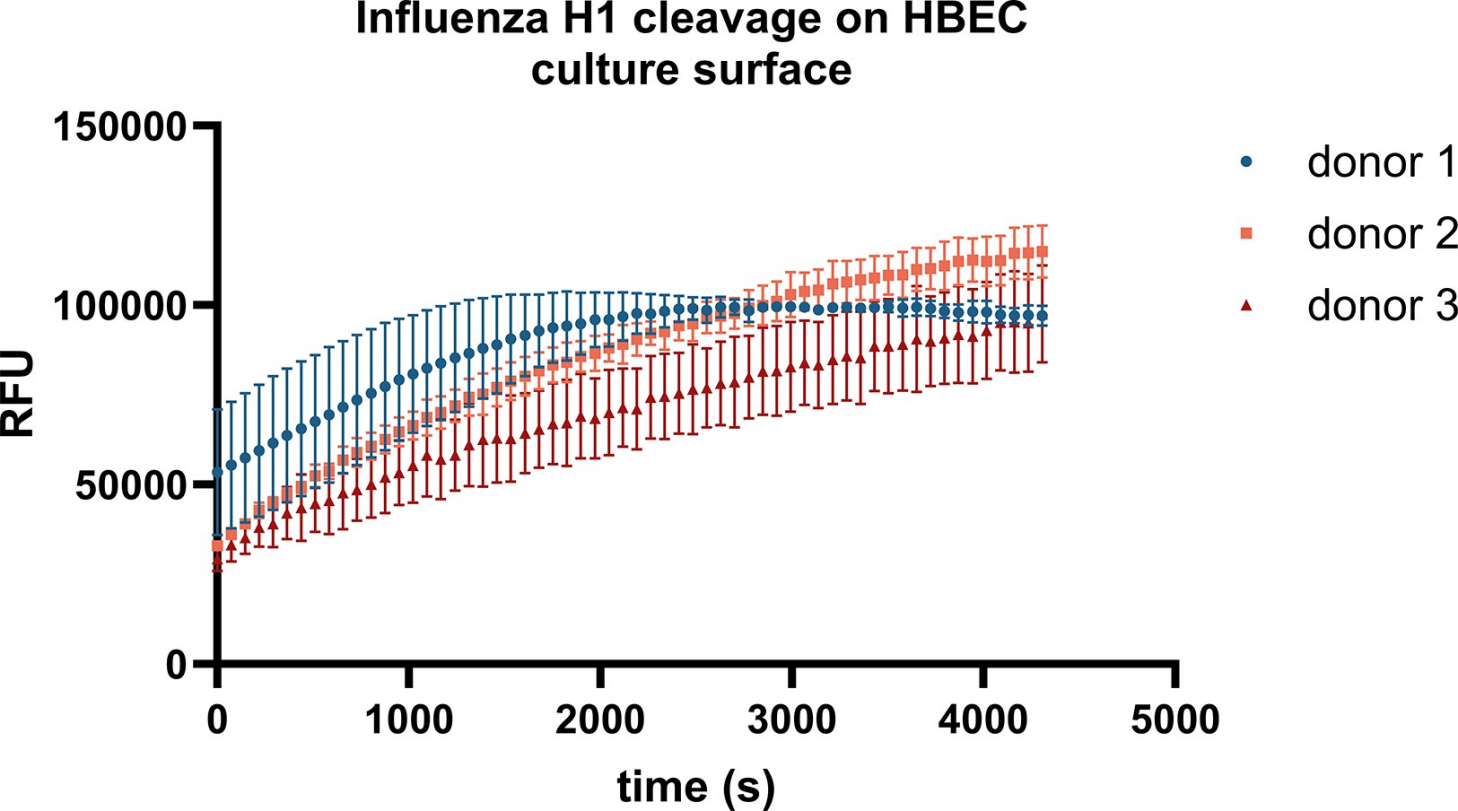
Proteolytic activity measured directly on the apical surface of ALI cultures. Proteolytic activity was measured on HBEC cultures at ALI. A 50 μM solution of Influenza H1 peptide in 100 μl of total volume was added to the apical surface of duplicate cultures from n = 3 (2M, 1F) donors. The prior apical wash of the cultures occurred 72h prior. Change in fluorescence intensity over time (s) on the surface of the cultures was then measured on a plate reader.

While the prior data were generated using apical washes, we also measured proteolytic activity directly on the surface of primary human epithelial cell cultures at air-liquid interface (ALI). Cleavage of the Influenza H1 peptide was measured on the apical surface of HBECs (n = 3, 2M, 1F)), demonstrating interindividual variability in rates of cleavage between donors (Fig 4).

### Proteolytic activity toward Influenza H1 can be measured in NLF and NELF

**Fig 5:**
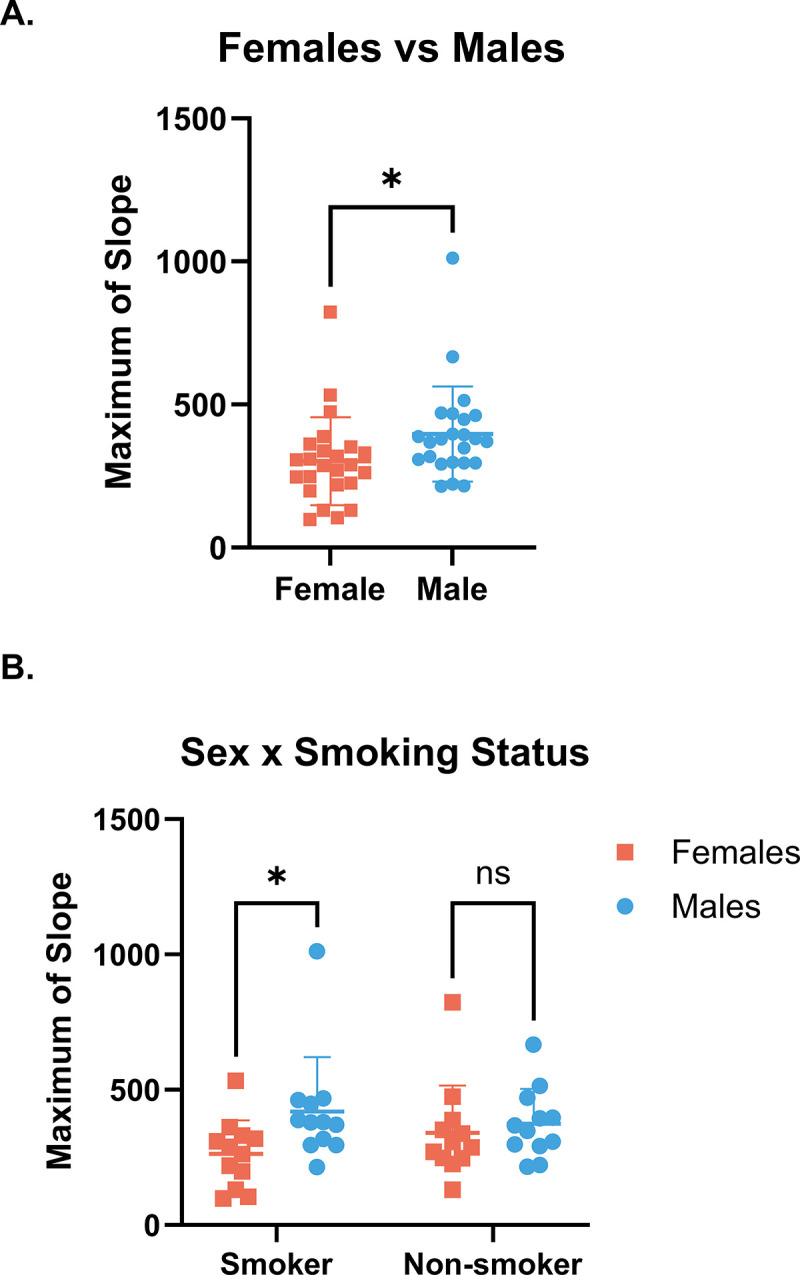
Differences in cleavage of Influenza H1 by proteases in NLF from male and female smokers. NLF samples were collected from (n = 48) smoking and non-smoking adults. The Influenza H1 peptide solution (50 μM) was then mixed with the samples and fluorescence intensity was measured on a plate reader. No outliers were identified by the ROUT (Q = 1%) method. A) Sex difference between n = 24 Male and n = 24 Female donors when the data are aggregated by smoking status (unpaired t-test, p≤0.05). B) Separating by smoking status demonstrates greater cleavage of the Influenza H1 peptide in samples from male smokers compared to female smokers, with no statistically significant difference between non-smokers (2-way ANOVA with Bonferroni‘s post hoc test, p≤0.05). In both plots, mean with standard deviation is shown.

**Fig 6:**
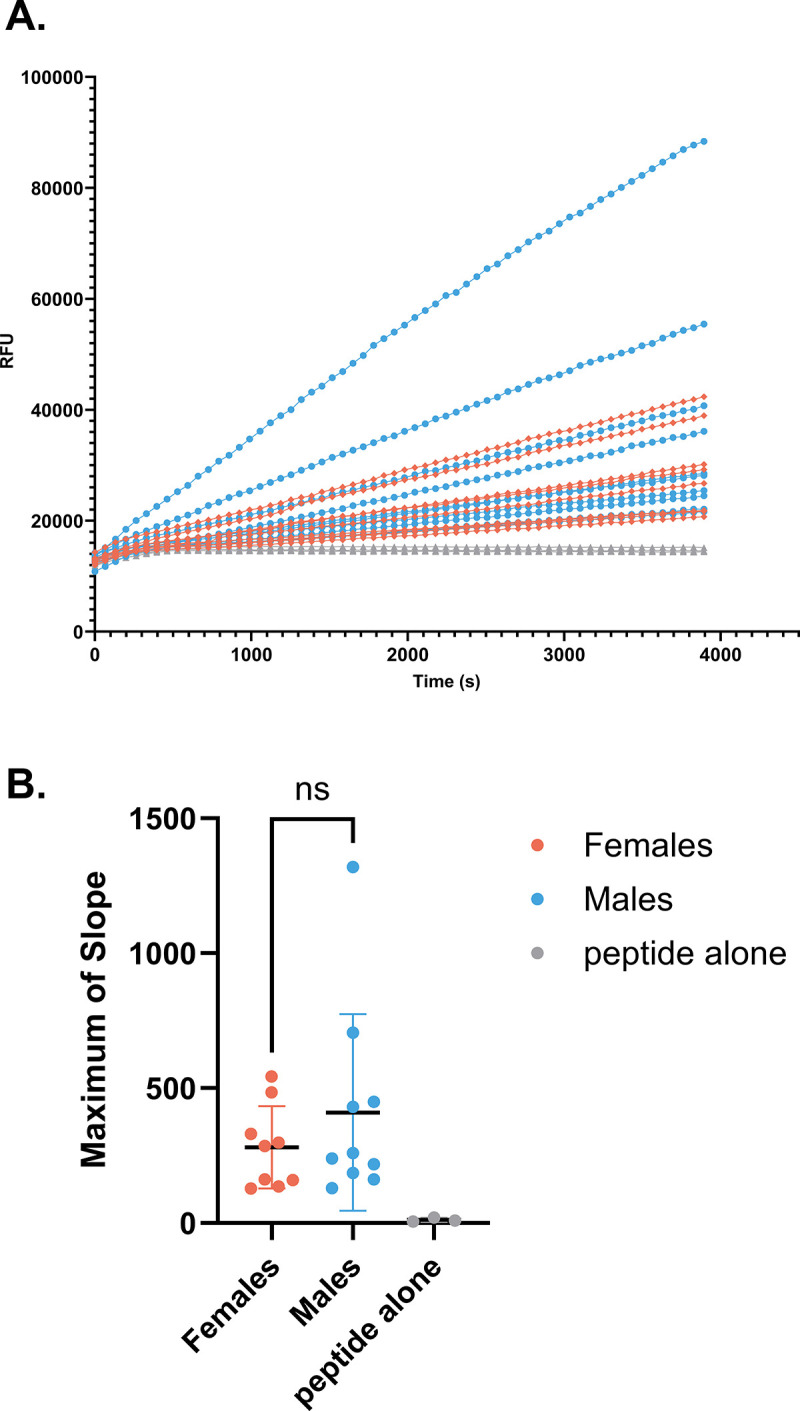
Proteases in NELF cleave Influenza H1. NELF samples were collected and mixed with the IQF Influenza peptide solution. Change in fluorescence intensity over time was then measured with a plate reader. A) Cleavage of the Influenza H1 peptide by proteases in NELF samples from n = 19 donors, males in blue (n = 10), and females in orange (n = 9). Triplicates of a peptide-only control (i.e. no NELF) are in light gray. B) Maximum rate of cleavage for all samples. Maximums of slope were calculated for data in the range of time = 480-3894s. Welch’s unequal variance *t*-test was used to evaluate difference in maximum slope between sexes.

To evaluate whether proteolytic activity towards Influenza virus from clinical samples could be detected by this assay *ex vivo*, we measured rates of cleavage of the viral peptides by nasal lavage fluid (NLF) samples collected from male and female smokers and non-smokers. As shown in Fig 5, the rate of cleavage of Influenza H1 peptide was higher in males compared to females in the aggregate dataset with both smokers and non-smokers. Stratification by smoking status revealed that cleavage of Influenza H1 was specifically elevated in male smokers (Fig 5B). There was no difference in rate of cleavage of Influenza H1 between non-smoking males and females and no difference between smokers and non-smokers. Similarly, we sought to demonstrate proteolytic capacity toward viral substrates in nasal epithelial lining fluid (NELF). Rates of cleavage of Influenza H1 was measured using NELF samples from male (n = 10) and female (n = 9) non-smoking donors. Change in fluorescence intensity versus time for each donor is plotted in Fig 6A. The maximum slope of the cleavage reactions by sex of donor is also shown (Fig 6B). While there was no statistically significant difference in maximum rate of cleavage between NELF from males and females, there is a large degree of interindividual variability in rate of cleavage between donors.

### ALI culture apical washes also cleave SARS-CoV S proteins

**Fig 7:**
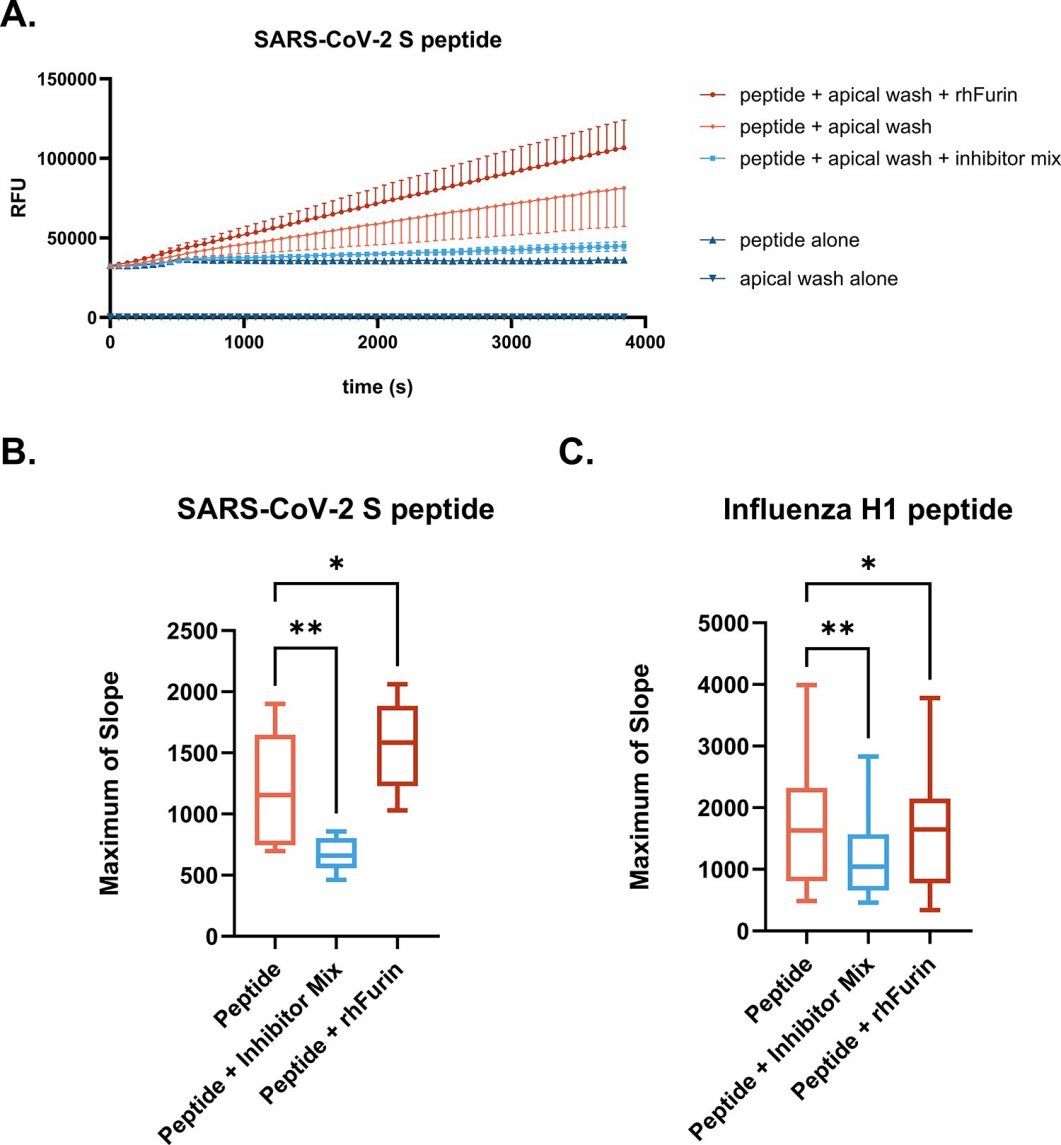
Cleavage of SARS-CoV-2 S IQF peptide by apical wash samples from HNECs. Cultures from n = 9 (5M, 4F) HNEC donors were apically washed. At the time of assay, samples were then mixed with the 50μM SARS-CoV-2 IQF peptide solution and the cleavage reaction was observed in a plate reader. A) Change in RFU vs time is shown in orange. The addition of a mixture of protease inhibitors (light blue) or rhFurin (red) on rate of cleavage is also shown. Controls (peptide with no sample or HNEC samples with no peptide) are in dark blue. Samples were collected 4 d since the prior apical wash. B) Maximum rates of reaction for SARS-CoV-2 S and C) for Influenza H1. Differences between groups were detected by repeated measures one-way ANOVA with Dunnett‘s post hoc test; * p≤0.05, ** p≤0.01.

**Fig 8:**
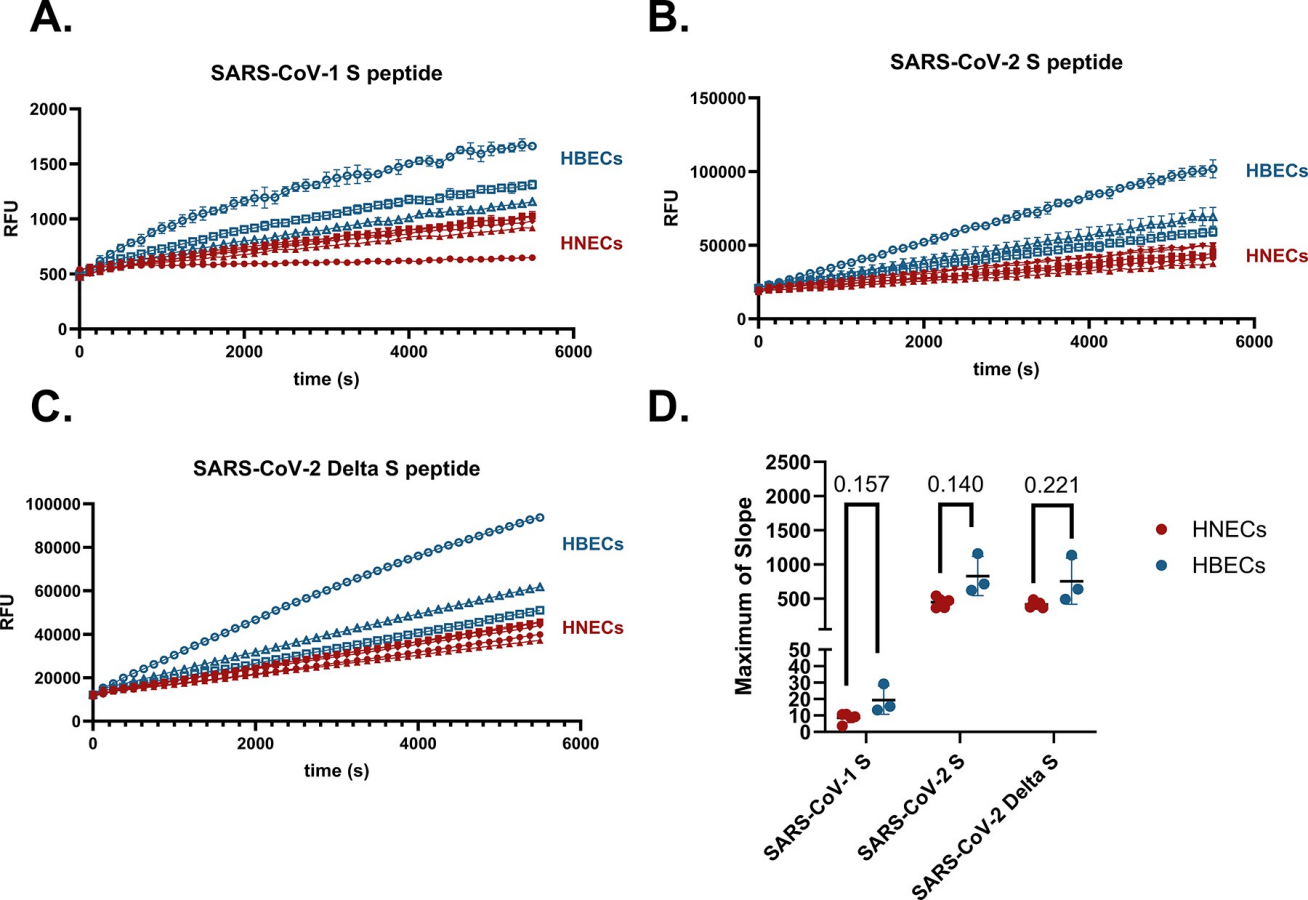
Proteases in HNEC and HBEC apical washes cleave SARS coronavirus peptides. Rates of cleavage of SARS S1/S2 IQF peptides by apical wash samples from n = 5 (3M, 2F) HNEC donors (in red) and n = 3 (2M, 1F) HBEC donors (in blue). Cleavage of A) SARS-CoV-1 B) SARS-CoV-2, and C) SARS-CoV-2 Delta. All samples were collected 7 d from the prior apical wash. Each line represents the mean of three technical replicates with standard deviation. D) Differences in maximum of slope between HBECs and HNECs for each peptide were tested with individual Welch’s unequal variance *t*-tests.

**Fig 9:**
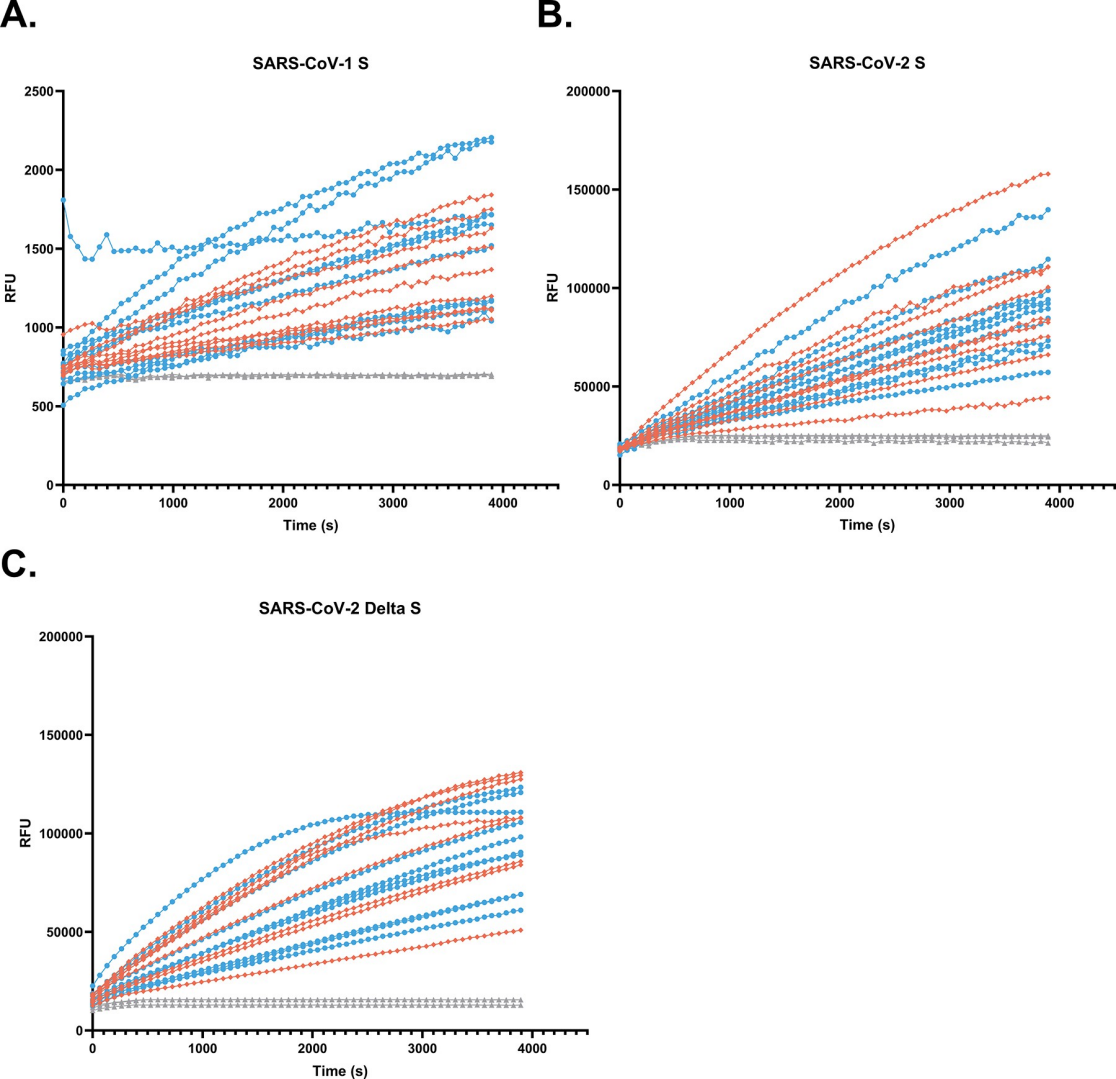
Proteases in NELF cleave SARS coronavirus peptides. Rate of cleavage of the SARS coronavirus peptides (50 μM) by proteases in NELF samples from n = 19 donors, males in blue (n = 10), and females in orange (n = 9). Peptide-only controls are shown in light gray. Cleavage of SARS-CoV-1 is shown in (A), SARS-CoV-2 in (B) and SARS-CoV-2 Delta in (C).

To demonstrate the utility of this assay to other viruses of public health relevance, we tested HNEC- and HBEC-mediated cleavage of the SARS-CoV peptides listed in Table 1. Fig 7A shows that enzymes in HNEC apical washes indeed cleave the SARS-CoV-2 S peptide, and addition of a protease inhibitor cocktail greatly decreases the maximum rate of cleavage of the peptide. Furthermore, addition of rhFurin to the SARS-CoV-2 S peptide reaction increases the rate of cleavage of this peptide (Fig 7B). In contrast, when rhFurin is added to the Influenza H1 cleavage reaction, there is a slight but statistically significant decrease in rate of peptide cleavage (Fig 7C). Furthermore, Fig 8A–8C demonstrate that proteases secreted from the apical surface of HNECs and HBECs grown at ALI successfully cleave the S peptides of SARS-CoV-1, SARS-CoV-2, and SARS-CoV-2 Delta, respectively. There was again no statistical difference in the maximum of slope between HNEC and HBEC cultures for any of the SARS-CoV peptides (Fig 8D). Cleavage of the SARS-CoV-1 S peptide occurred at a much lower rate than for the other peptides. Fig 9 demonstrates proteases in nasal epithelial lining fluid (NELF) from the same donors used for the influenza H1 cleavage assay also cleave SARS-CoV-1, SARS-CoV-2, and SARS-CoV-2 Delta S peptides. Similar to influenza H1, there is much interindividual variability in cleavage of these peptides.

## Discussion

We sought to better understand the proteolytic landscape present on the airway surface and demonstrate the utility of a simplified method for evaluating the overall proteolytic capacity of airway samples which does not rely on measurement of individual proteins. In airway surface liquid samples from primary *in vitro* airway cultures as well as clinical *ex vivo* samples, we identified numerous proteases and antiproteases with diverse but overlapping substrate specificities. Additionally, a method for efficiently probing proteolytic activity (in this case toward viral substrates) was presented, revealing active proteases can be easily measured in a variety of airway samples. Furthermore, these findings reveal interindividual differences in cleavage and activation of multiple airway viruses, possibly indicating differential susceptibility to infection. To demonstrate an application of the assay, cleavage of Influenza H1 peptide was compared in NLF samples from male and female smokers and non-smokers, revealing NLF from male smokers more readily cleaved Influenza H1 compared to female smokers.

The surfaces of the airways exemplify a complex proteolytic landscape populated with diverse and mechanistically redundant proteases and antiproteases, demonstrated in Tables 5 and 6. Previous studies have identified differentially expressed proteases or antiproteases in the proteomes of airway samples from healthy versus disease phenotypes [66, 67]. However, to our knowledge, this study represents the first time specific proteomic profiling of both airway proteases and antiproteases has been assessed in airway samples from healthy donors. Our analysis puts a particular emphasis on proteases/antiproteases in the upper airway mucosa, which represents the initial target for many respiratory viruses. We identified 48 unique proteases in the HNEC apical wash samples and 57 in the NELF samples, which is in contrast to a former study which assessed the protease composition of bronchoalveolar lavage fluid from healthy donors and identified only 13 proteases [9]. Moreover, this study compares the secreted proteases and antiproteases detected from *in vitro* primary nasal epithelial cells and *ex vivo* nasal epithelial lining fluid (NELF) from healthy human donors. Unsurprisingly, there were greater numbers of unique proteases and antiproteases detected in the NELF samples compared to the HNEC apical washes, which could be explained by the presence of cell types other than epithelial cells in the human nasal mucosa *in vivo*, including monocytes and neutrophils. However rather surprisingly, there were some proteases detected in HNEC apical wash samples which were not detected in NELF, including matriptase (ST14) and several metalloproteases (ADAM9, ADAM10, ADAM28). While this could reflect interindividual differences in nasal protease expression (since these samples were obtained from different donors), changes in cell biology induced from growing cells in culture could also provide an explanation. Although the data were not obtained from matched donors, we believe that the results are impactful in illustrating the complexity of the whole proteolytic environment found in this region of the airway. Further, these data emphasize the difficulty of predicting overall changes in the proteolytic environment of the airway based on changes in single enzymes or inhibitors, a method which has been previously used to predict changes in infection susceptibility. Unexpectedly, TMPRSS2 was not detected in the *in vitro* or *ex vivo* airway samples. High levels of expression of this protease in nasal epithelial cells has been reported previously [68]. Other transmembrane serine proteases (TMPRSS11D and TMPRSS11E) were detected in our samples. The lack of detectable TMPRSS2 in our samples may indicate lower extracellular release of this protein compared to the other TMPRSS proteins but this should be investigated in future work.

Assaying proteolytic capacity of airway samples toward substrates of interest presents an effective method of detecting perturbations of the protease:antiprotease ratio following environmental exposures or therapeutic treatments and in the case of underlying diseases. The assay we show here offers advantages over methods employing infectious viruses and Western blot to assess how changes in the proteolytic environment impact susceptibility to viral infection. Our data demonstrate that the assay developed and optimized here has utility in examining the proteolytic capacity of apical secretions from HNEC and HBEC organotypic cultures to activate multiple respiratory viruses of public health relevance (Figs 1, 3, 4, 7, and 8). Additionally, because of their immediate human relevance, we demonstrate proteolytic activity in clinical human airway samples. We found that proteolytic activity can indeed be measured in stored nasal samples (Figs 5, 6, and 9). Our data do not elucidate which proteases are responsible for cleaving the peptides, though the proteomic data indicate that the proteolytic milieu of these samples is complex, owing to the large diversity in proteases there. Based on Figs 1 and 7 it is apparent that a mix of serine and cysteine proteases (and furin-like proteases in the case of SARS-CoV-2) are active in cleaving the viral peptides. However, these data also show interindividual variability in cleavage efficacy of the peptide substrates. Thus, determining which proteases contributed to cleavage likely depends on each individual’s overall protease and antiprotease composition. Since equal substrate concentrations and sample volumes were used across each experiment, our results suggest that differences in expression of proteases (or antiproteases) in the extracellular space drives the interindividual differences observed. While it is also possible that individuals differ in the activities of their proteolytic enzymes, based on the proteomic data it seems most likely that the overall protease:antiprotease milieu differs among donors. This notion is supported by the variability in cleavage rates between donors observed in Fig 3 despite these samples being normalized by total protein concentration. The clinical NELF samples further varied significantly by donor in rate of cleavage of all viral peptides (Figs 6 and 9).

Because the assay provides an overall glimpse at proteolytic activity toward a substrate of interest, it may have utility in identifying potentially susceptible populations. We have previously demonstrated that markers of viral replication are enhanced in the nasal mucosa of people who smoke or are routinely exposed to secondhand smoke [56]. Smoking has been found to increase expression of proteases such as neutrophil elastase, TMPRSS2, and certain matrix metalloproteases in the lungs [45, 69, 70]. However, smoking also increases antiprotease SLPI expression [47]. Thus, we evaluated the effects of smoking status on cleavage of influenza H1 using NLF samples. We found no difference in cleavage of the Influenza H1 substrate in NLF samples from smokers and non-smokers (Fig 5). Previous work from our group has shown that sex differences arise in responses to influenza infection following exposure to woodsmoke [52, 71]. In the present study, after disaggregating the data by sex we report greater proteolytic activation of Influenza H1 in NLF from male smokers compared to female smokers (Fig 5), suggesting that sex and exposure may together impact susceptibility to infection.

Additionally, our data suggest that proteases accumulate on the apical surface of ALI cultures over time and routine washing of these cultures removes proteases from the culture surface. This may be an important consideration for studies involving inoculation of the apical surface of cultures with respiratory viruses or evaluating efficacy of inhaled therapeutics.

With access to a plate reader with an atmospheric control unit, this assay can also be used to measure proteolytic cleavage directly on the apical surface of ALI cultures in real time, demonstrated in Fig 4. This expansion of our assay also allows the assessment of membrane-bound/tethered proteases in the respiratory mucosa. Dosing test compounds in the basolateral medium presents an application of the assay in testing efficacy of drugs designed for systemic circulation to modulate the protease:antiprotease balance on the airway surface.

An important limitation of the work presented here is the use of small peptides to examine proteolytic activity in our samples rather than native viral fusion proteins or viruses. Cleavage efficacy of proteolytic enzymes is not dependent solely on the amino acid sequence immediately preceding the cleavage site but also on the three-dimensional shape and physicochemical properties of the substrate protein. Additionally, during synthesis of progeny virions in a host cell, depending on localization of the virions, local proteases can cleave the fusion protein before it ever enters the extracellular space. Though we have validated that extracellular proteases released apically by HNECs activate native Influenza virus HA during infection [46], we have not validated SARS-CoV-2 S cleavage in the same manner due to its high pathogenicity and tight regulation surrounding its use. Based on the large number of unique proteases observed in our *in vitro* and *ex vivo* airway samples, many of which have been reported to cleave influenza H1 and coronavirus S proteins, we present this assay as a straightforward method of preliminarily assessing proteolytic activity in a variety of airway sample types. A further limitation which should be highlighted is the lack of a quantitative measure of proteolysis in our samples or standard for comparison; rather this assay reflects relative proteolytic cleavage rates in a sample set toward with a specific substrate. As discussed above, using a robust sample size of n = 19 healthy donors, we observed a wide range of natural variability within the population of proteolytic activity of nasal secretions (Figs 6 and 9). Additionally, through other observations presented in this study, we showed that inhibition of proteases using inhibitors decreases the rate of cleavage of the peptide substrates and increasing substrate concentration increases rates of cleavage, showing specificity. However, a limitation of the data presented here is the use of a protease inhibitor cocktail, which does not allow for assessing the contribution of the individual inhibitors in the decreased cleavage rates observed here.

The Spike protein of SARS coronaviruses undergoes two cleavage events to fuse with the host membrane; one at the S1/S2 site and another at the S2’ site [26]. Similar to Influenza A, a variety of extracellular and membrane-bound host proteases, including human airway trypsin (HAT), cathepsin B and L, TMPRSS2, and matriptase, activate the Spike proteins of SARS-CoV-1 and SARS-CoV-2 [36–39]. However, only HAT, matriptase, and cathepsin B/L have been shown to cleave SARS Spike at the S1/S2 site, and only in *in vitro* experimental systems [37, 38]. In our assay, a mixture of chemical protease inhibitors against serine, cysteine, and Furin-like proteases reduced the cleavage rate of the SARS-CoV-2 peptide (which models the S1/S2 site) more dramatically than the Influenza H1 peptide in the same samples (Fig 7). This suggests that at least partially different subsets of proteases cleave these peptides. The increased rate of cleavage of the SARS-CoV-2 S peptide upon addition of rhFurin is expected since this virus acquired a Furin cleavage site in the S protein, characterized by multiple basic amino acids (typically Arginine) grouped together (underlined in Table 1) [72, 73].

Influenza H1 does not contain a Furin cleavage site and addition of recombinant Furin slightly reduced the rate of cleavage of the Influenza H1 peptide. One possible explanation for this finding is competitive binding by the rhFurin to the peptide without catalysis of the cleavage reaction, reducing interactions between the peptide and proteases which successfully cleave it. Although the SARS-CoV-2 S1/S2 cleavage site has been previously shown to be cleaved by extracellular and membrane-bound proteases [37], it is likely that this site is primarily cleaved by intracellular Furin before progeny virions are released from an infected cell during biosynthesis of the S protein [26]. Thus, modeling activation of this cleavage site by airway surface liquid samples represents a limitation of this study. An IQF peptide modeling the S2’ site may be a more relevant substrate for extracellular proteases in the airway and should be examined in future work.

As shown in Table 1, the SARS-CoV-2 S protein contains two additional basic amino acids adjacent to its S1/S2 cleavage site (termed a “multibasic” cleavage site) while SARS-CoV-1 S contains only one basic amino acid at the cleavage site (a “monobasic” cleavage site) [73]. The multibasic SARS-CoV-2 cleavage site confers an active site for Furin and Furin-like proteases, which have been found to cleave the S1/S2 site of SARS-CoV-2 much more effectively than SARS-CoV-1 [37, 73]. Multibasic cleavage sites in hemagglutinin (HA) are also a characteristic of highly pathogenic avian influenza strains, but not of low-pathogenicity strains [28]. Our assay agrees with prior findings in that we observed a much lower cleavage rate of the SARS-CoV-1 peptide compared to the SARS-CoV-2 peptide (Figs 8 and 9).

The SARS-CoV-2 Delta variant contains a P681R mutation conferring an additional basic amino acid in the S1/S2 cleavage site (see Table 1). In prior studies, the P681R mutation in infectious SARS-CoV-2 viruses conferred enhanced S1/S2 cleavage of the Spike protein [74, 75]. In our study, however, we observed no statistically significant difference in rates of cleavage between the Delta and wild-type SARS-CoV-2 peptides in our samples. We hypothesize our observation is due to the absence of tertiary structure which accurately reflects the cleavage site of the native Spike protein in our IQF peptides. This represents an inherent limitation of using a small peptide to model this enzyme-substrate interaction.

## Conclusions

The data shown here reinforce the vast diversity of unique proteases and antiproteases found in airway surface liquid samples from both organotypic airway culture models and clinical human samples. The methodology we describe is a straightforward, easy to use, and adaptable approach for measurement of the proteolytic activity of airway samples toward viral substrates, though the peptide design could be modified for any substrate of interest. This assay has utility for assessment of environmental exposures, disease states, or pharmaceutical interventions on the activity of airway proteases used by respiratory viruses to initiate infection. In addition, this methodology offers the ability to upscale to medium/high throughput and has greater practicality compared to measurement of cleavage rates by individual proteases or assessment of concentrations of individual proteases/antiproteases, which offer only an approximation of total proteolytic activity.

## Supporting information

S1 Fig Proteases accumulate on the apical surface of ALI cultures over time. The apical surfaces of HNEC cultures from n = 5 (3M, 2F) donors were washed at 24, 48, 72, or 96 h post an initial wash and rate of cleavage of the influenza H1 IQF peptide was measured in each wash sample. Briefly, the samples were mixed with the IQF peptide and rate of cleavage was calculated from the change in fluorescence intensity in the sample over time, read in a microplate reader.
(TIF)

S2 Fig Proteases remain enzymatically active with multiple freeze/thaws. Effects of 0–4 freeze and thaw cycles of apical wash samples from n = 5 (3M, 2F) HNEC donors on proteolytic activity toward the Influenza H1 peptide. Lines represent individual donors and bars are means of all 5 biological replicates. There were no statistically significant differences between groups by repeated measures one-way ANOVA with Bonferroni’s post hoc test.
(TIF)
