# Supplementary figures and images for: Profiling endogenous airway proteases and antiproteases and modeling proteolytic activation of Influenza HA using in vitro and ex vivo human airway surface liquid samples
Source: PLoS One. 2024 Dec 31;19(12):e0306197. doi: 10.1371/journal.pone.0306197 (PMC11687774; doi:10.1371/journal.pone.0306197)

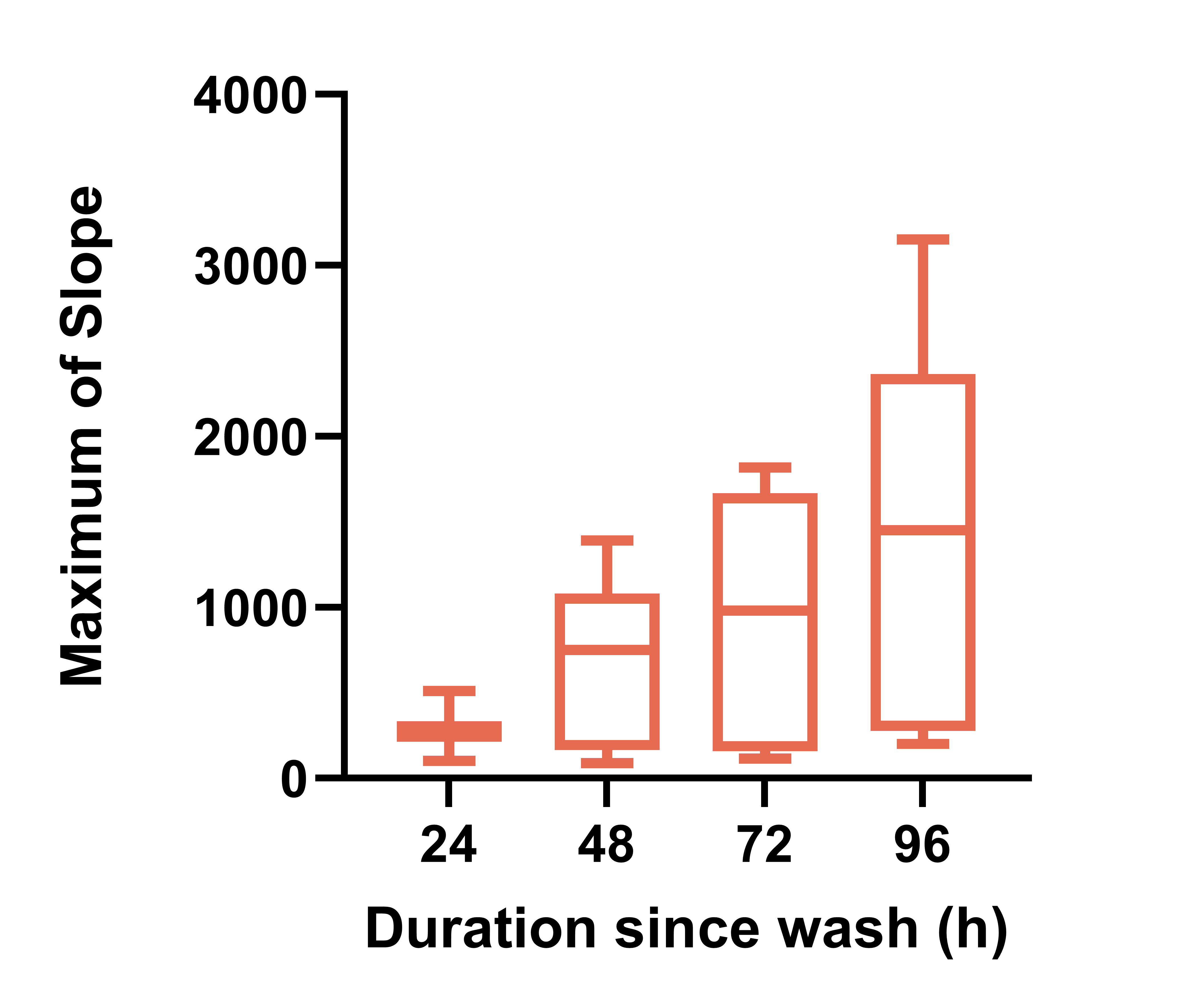

Supplement: S1 Fig — The apical surfaces of HNEC cultures from n = 5 (3M, 2F) donors were washed at 24, 48, 72, or 96 h post an initial wash and rate of cleavage of the influenza H1 IQF peptide was measured in each wash sample. Briefly, the samples were mixed with the IQF peptide and rate of cleavage was calculated from the change in fluorescence intensity in the sample over time, read in a microplate reader. (TIF) [file pone.0306197.s001.tif]

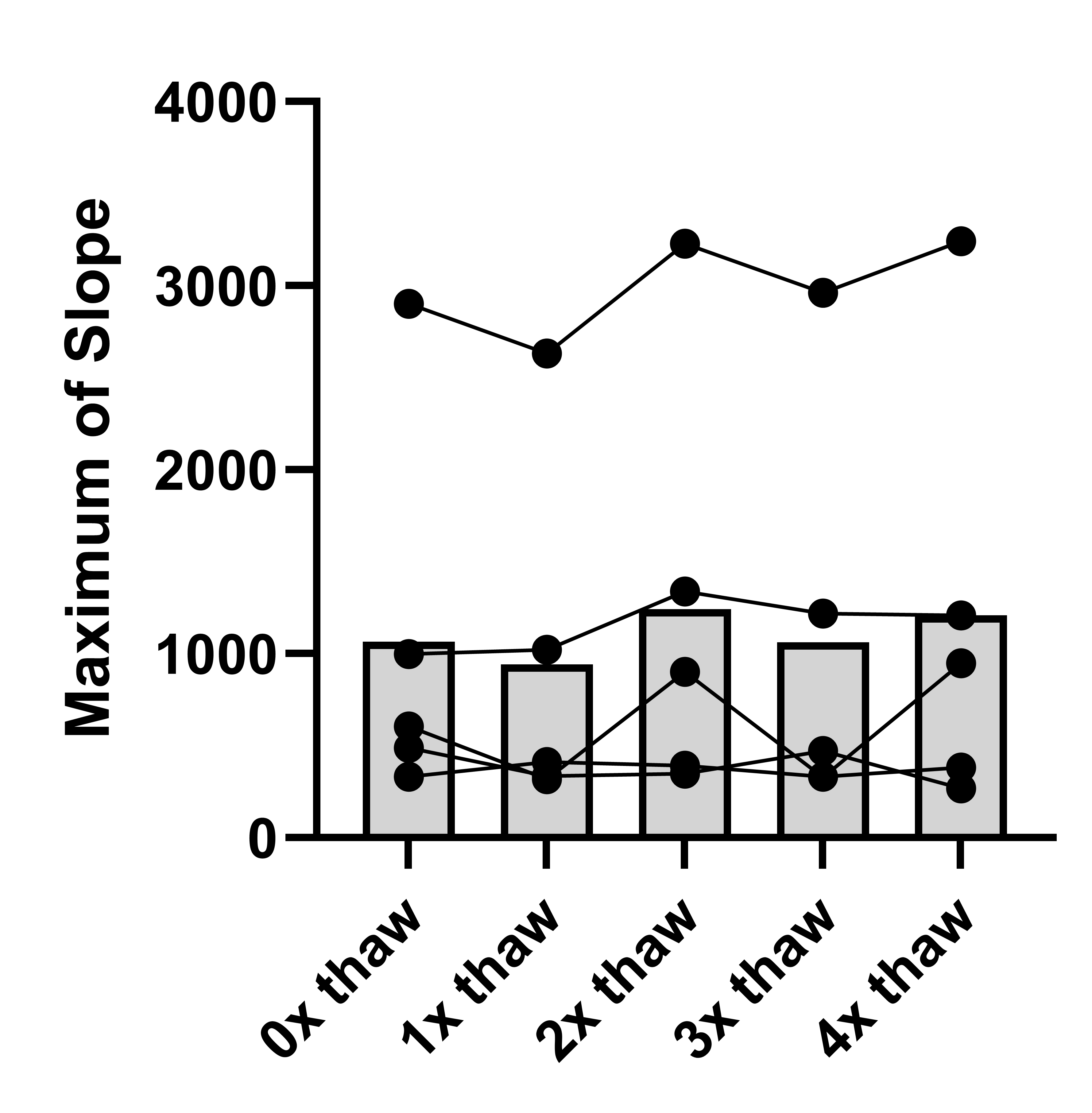

Supplement: S2 Fig — Effects of 0–4 freeze and thaw cycles of apical wash samples from n = 5 (3M, 2F) HNEC donors on proteolytic activity toward the Influenza H1 peptide. Lines represent individual donors and bars are means of all 5 biological replicates. There were no statistically significant differences between groups by repeated measures one-way ANOVA with Bonferroni’s post hoc test. (TIF) [file pone.0306197.s002.tif]
